# HSA^LR^ Mice Exhibit Co-Expression of Proteostasis Genes Prior to Development of Muscle Weakness

**DOI:** 10.3390/ijms262110793

**Published:** 2025-11-06

**Authors:** Dusan M. Lazic, Vladimir M. Jovanovic, Jelena Karanovic, Dusanka Savic-Pavicevic, Bogdan Jovanovic

**Affiliations:** 1 Center for Human Molecular Genetics, Faculty of Biology, University of Belgrade, Studentski Trg 16, 11000 Belgrade, Serbia; dusan.lazic@bio.bg.ac.rs (D.M.L.); duska@bio.bg.ac.rs (D.S.-P.); 2 Department of Biology, Chemistry and Pharmacy, Freie Universität Berlin, Königin-Luise-Str. 1-3, 14195 Berlin, Germany; vladimir.jovanovic@bio.bg.ac.rs; 3 Gene Regulation in Cancer Group, Institute of Molecular Genetics and Genetic Engineering, University of Belgrade, Vojvode Stepe 444A, 11042 Belgrade, Serbia; jelena.karanovic@imgge.bg.ac.rs

**Keywords:** myotonic dystrophy type 1, repeat expansions, HSA^LR^, WGCNA, protein homeostasis, proteostasis, ER-associated degradation, ubiquitin-proteasome system, autophagy, mitophagy

## Abstract

Myotonic dystrophy type 1 (DM1) is a progressive multisystemic disease caused by a CTG repeat expansion in the *DMPK* gene. The toxic mutant mRNA sequesters MBNL proteins, disrupting global RNA metabolism. Although alternative splicing in DM1 skeletal muscle pathology has been extensively studied, early-stage transcriptomic changes remained uncharacterized. To gain deeper and contextual insight into DM1 transcriptome, we performed the first Weighted Gene Co-expression Network Analysis (WGCNA) on skeletal muscle RNA sequencing data from the widely used DM1 mouse model HSA^LR^ (~250 CTG repeats). We identified 532 core genes using data from 16-week-old mice, an age before the onset of muscle weakness. Additional differential expression analysis across multiple HSA^LR^ datasets revealed 42 common up-regulated coding and non-coding genes. Within identified core genes, the pathway gene-pair signature analysis enabled contextual selection of functionally related genes involved in maintaining proteostasis, including endoplasmic reticulum (ER) protein processing, the ubiquitin-proteasome system (UPS), macroautophagy and mitophagy, and muscle contraction. The enrichment of ER protein processing with prevailing core genes related to ER-associated degradation suggests adaptive chaperone and UPS activation, while core genes such as *Ambra1*, *Mfn2*, and *Usp30* indicate adaptations in mitochondrial quality control. Coordinated early alterations in processes maintaining protein homeostasis, critical for muscle mass and function, possibly reflect a response to cellular stress due to repeat expansion and appears before muscle weakness development. Although the study relies exclusively on transcriptomic analyses, it offers a comprehensive, hypothesis-generating perspective that pinpoints candidate pathways, preceding muscle weakness, for future mechanistic validation.

## 1. Introduction

Myotonic dystrophy type 1 (DM1, OMIM: #160900) is the most common adult-onset muscular dystrophy. It is characterized by multisystemic symptoms whose progression cannot be slowed or stopped with available treatments. The underlying autosomal dominant mutation is an unstable expansion of 50 to more than 1000 CTG repeats in the 3′ untranslated region of the DM1 protein kinase (*DMPK*) gene [1]. Disease severity is broadly correlated to the variable number of repeats [2]. Germline repeat instability underlies genetic anticipation, causing symptoms to start earlier and worsen in later generations, while somatic repeat instability drives disease progression [3]. The main mechanism of DM1 molecular pathogenesis is RNA gain-of-function, where *DMPK* transcript with expanded CUG repeats acquires new toxic functions [4]. In DM1, tissue and developmental stage-specific splicing is disrupted by the toxic RNA inactivation of MBNL proteins by sequestration in ribonuclear foci and downstream overactivation of CELF1 protein by hyperphosphorylation [5]. Along with the aberrant splicing as a molecular hallmark of DM1, the toxic RNA also causes disruptions in polyadenylation, transcription, and translation of numerous genes [6].

The development of various genetically engineered mouse models has played an essential role in molecular, cellular, pathophysiological, and preclinical research of DM1. HSA^LR^ model is the most commonly used one and was generated by random insertion of a genomic fragment carrying the human skeletal actin gene (*ACTA1*) with ~250 CTG repeats in its 3′UTR [7]. This transgene is expressed only in skeletal muscles and its resulting transcript is retained in the nucleus [7]. These mice develop myotonia at 4 weeks of age, while at that time there are no signs of abnormal muscle histology [7]. Later, DM1-specific muscle histopathology is developed involving abundant central nuclei, ring fibers, and variable fiber size with no necrosis [7], whereas muscle weakness becomes obvious in adult mice at 24 weeks of age [8], and mortality increases by 41% by 44 weeks [9].

Transcriptomic studies on HSA^LR^ and *Mbnl* knockout mice, also serving as DM1 models, reveal commonalities in alternative splicing and differential gene expression [10,11]. Further, analysis of both transcriptome and proteome of HSA^LR^ mice has revealed a certain quantitative agreement of differentially expressed genes (DEGs) and alternative splicing [12]. While alternative splicing and DEG studies revealed important transcriptomic alterations in DM1, they offer limited insight into coordinated gene expression changes. Gene co-expression analysis enables the discovery of such groups of genes, highlighting system-level dysregulation. Yet, no gene co-expression networks were made for DM1 mouse models. Here, we performed an exploratory, comprehensive transcriptome analysis including weighted gene co-expression network analysis (WGCNA), to identify clusters of highly co-expressed genes associated with the HSA^LR^ mouse genotype and expected to be related with impaired molecular and cellular processes appearing downstream from the toxic RNA.

## 2. Results

The raw reads derived from 59 skeletal muscle samples of HSA^LR^ mice from seven bulk paired-end RNA-seq experiments were acquired from NCBI (BioProject database Accession Numbers and references to publications are given in Table S1). After raw reads preprocessing, the mapping rates ranged from 78 to 98%, while the percentage of reads successfully assigned to genes (gene quantification) ranged from 65 to 86% (Table S1).

WGCNA was performed to identify gene co-expression networks, representing modules of genes with potentially common biological functions, associated with DM1 across a set of samples [13]. The unsigned network type (which uses absolute values of correlations to cluster co-expressed genes) was chosen to provide robust representation of gene co-expression patterns. The Hicks et al. dataset (PRJNA1103789) [14] was selected for WGCNA because it contains the largest number of samples from adult HSA^LR^ (9 samples, 16 weeks old) and wild-type mice (9 samples, 15 weeks, and 6 days old) from both proximal and distal skeletal muscles (Table S1).

**Figure 1:**
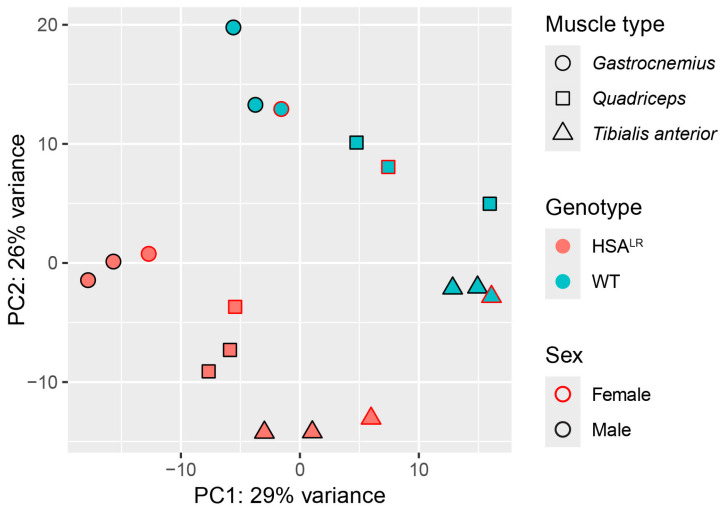
Principal component analysis (PCA) illustrates the effect of genotype and muscle type on detected gene expression patterns in Hicks et al. dataset (PRJNA1103789). PCA plot performed on variance-stabilized counts demonstrates that the main drivers of separation between samples are genotype followed by muscle type. The grouping of samples was not influenced by sex.

First, a principal component analysis (PCA) of the normalized (variance-stabilized) counts was performed to explore the sample clustering according to gene expression. It was found that genotype (HSA^LR^ or wild type) had the strongest influence on the separation of samples, followed by muscle type (*quadriceps*, *tibialis anterior*, or *gastrocnemius*), with no effect of sex (Figure 1). This is in line with functional measurements, histology, and molecular/biochemical profiles between male and female HSA^LR^ mice that showed no significant differences [15]. Similarly, samples displayed clustering based on distances in a hierarchical clustering step of WGCNA (Figure S1A).

**Figure 2:**
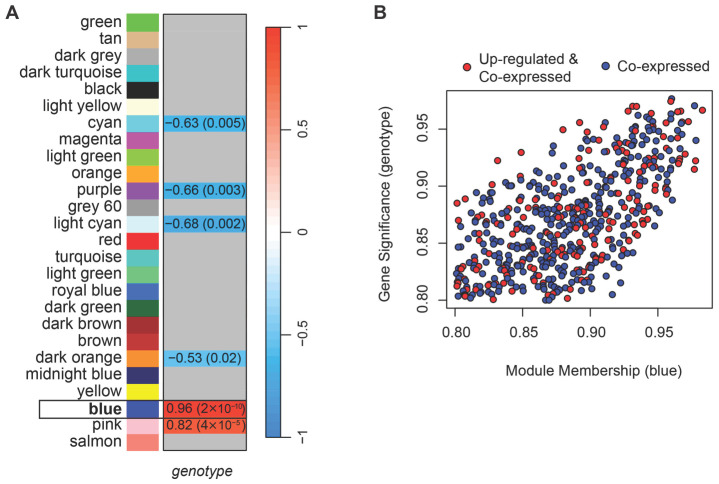
Weighted Gene Co-expression Analysis (WGCNA) shows 26 modules of co-expressed genes, with the blue module (framed) showing the strongest association with HSA^LR^ genotype. (**A**) Heatmap showing Pearson correlation between module eigengenes, i.e., the first principal components of modules, and genotype (HSA^LR^ vs. wild type), with corresponding *p*-values in parentheses. Two module eigengenes (blue and pink) show significant positive correlations with the HSA^LR^ genotype, and four modules (cyan, purple, light cyan, dark orange) show significant negative correlations. Gray boxes indicate correlations with *p*-value > 0.05. (**B**) Scatterplot of gene significance (GS) versus module membership (MM) for genes in the blue module. Shown genes are with GS and MM > 0.8 and their *p*-values ≤ 0.05. Red points mark genes that are also up-regulated in HSA^LR^ according to DESeq2 analysis of Hicks et al. dataset (PRJNA1103789), indicating overlap between co-expression and differential expression. These high-MM, high-GS genes are hereafter referred to as core genes of the blue module.

The WGCNA performed on normalized counts identified 26 modules of co-expressed genes (Figure S1B). A soft-thresholding power of four was selected as the lowest value at which the network approximately fits a scale-free topology (R^2^ > 0.8; Figure S2). Among the detected modules, two eigengenes, i.e., first principal components of the module (blue and pink) were positively correlated, and four (cyan, purple, light cyan, and dark orange) negatively correlated with the HSA^LR^ genotype (Figure 2A). For each gene, we calculated gene significance (GS), defined as the Pearson correlation between gene expression and the HSA^LR^ genotype (coded as 1 for HSA^LR^ and 0 for wild type), and module membership (MM), defined as the correlation between gene expression and the module eigengene. A threshold of 0.8 for the absolute values of both GS and MM was used together with *p*-values ≤ 0.05, to identify genes strongly associated with both the trait and their module. Only the blue module contained genes that met these criteria.

To further isolate genes driving the blue module’s positive correlation in HSA^LR^, we filtered for those with positive GS and positive MM above 0.8 (with corresponding *p*-values ≤ 0.05). Although the network was unsigned, focusing on genes with both positive GS and MM allowed us to identify a coherent subset of genes that are not only highly connected within the module but can also be more highly expressed in the HSA^LR^ group. These genes are likely to be presumed drivers of the module’s association with HSA^LR^ genotype (Figure 2B, Table S2) and were selected for downstream analyses (hereafter referred to as **core genes**). While Hicks et al. reported muscle type-dependent expression of the transgene [14], we did not observe significant associations of individual gene expression levels with muscle type in our data (|GS & MM| > 0.8, *p* ≤ 0.05).

**Figure 3:**
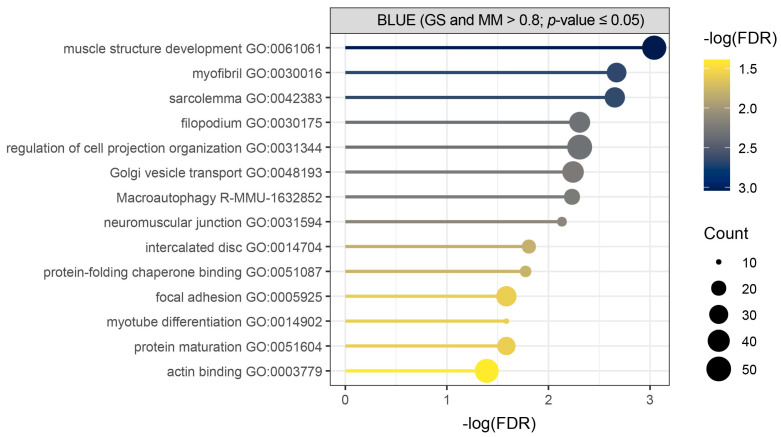
Over-representation analysis (ORA) performed for core genes of the blue module shows initial biological overview guiding towards muscle development, muscle cell components, macroautophagy, and protein folding and maturation. Metascape enrichment results of core genes (GS and MM > 0.8; *p*-value ≤ 0.05) detected in Hicks et al. dataset (PRJNA1103789) shows terms with (−log(FDR) > 1.3), where count represents the number of query genes in each enriched term.

Over-representation analysis (ORA) was used as an initial approach to identify enriched terms and pathways among the core genes of the blue module, applying a false discovery rate (FDR) threshold of 0.05 (Figure 3, Table S3). It showed significant enrichment of biological process terms related to muscle structure development, muscle cell components, macroautophagy, and protein folding and maturation in our query of total of 532 detected core genes (Figure 3, Table S3).

**Figure 4:**
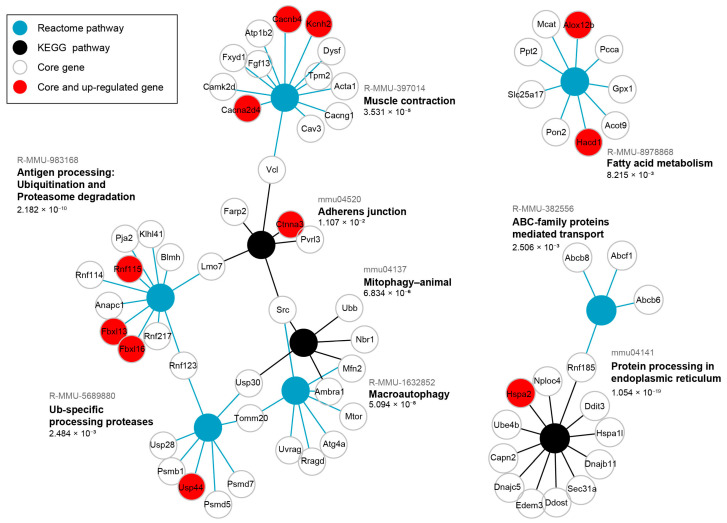
Significant over-representation (SIGORA) of blue module core genes, strongly associated with HSA^LR^ genotype, shows their convergence on proteostasis and muscle contraction. Graph representation of SIGORA Reactome and KEGG pathway gene-pair signature enrichment for core genes detected in Hicks et al. dataset (PRJNA1103789) that are strongly correlated with the blue module eigengene and HSA^LR^ genotype (GS and MM > 0.8, *p*-value ≤ 0.05), filtered by Bonferroni FDR < 0.05. The up-regulated were detected by DESeq2 in the same dataset and are present in the module (depicted by red). FDR values for each enriched pathway are written below, while pathway IDs are written in gray above the pathway names.

As many genes show a pleiotropic effect and take part in many functions, we additionally performed a significant over-representation analysis (SIGORA) of pathway gene-pair signatures [16] for the same core genes of the blue module. This analysis considers multiple genes as indicators of a certain pathway enabling a more contextual approach than ORA which treats pathways as simple sets of equally important individual genes and can lead towards too general hits when pathways share key components with more relevant processes. Significant results (FDR < 0.05) are shown in form of a graph (Figure 4) where there are different types of nodes representing each enriched pathway and enriched core genes. This analysis identified several signature pathways, reinforcing the major functional themes of blue module core genes.

Protein processing in ER is the enriched term with the lowest FDR (1.054 × 10^−19^, Figure 4). It encompasses DnaJ heat shock family (Hsp40) members (*Dnajc5*, *Dnajb11*), heat shock 70 family members (*Hspa1l*, *Hspa2*), along with ubiquitination-related genes (*Rnf185*, *Nploc4*, *Ube4b*). The coordinated expression of these genes, along with a protein that accelerates degradation of misfolded glycoproteins in the ER (*Edem3*) suggests the activity of ER-associated degradation (ERAD). A multifunctional transcription regulator *Ddit3*, involved in ER stress response, is an additional core gene in the term Protein processing in ER. Next, the term Antigen processing Ubiquitination and Proteasome degradation (FDR = 2.182 × 10^−10^), contains E3 ubiquitin-ligase genes (*Fbxl13*, *Fbxl16*, *Pja2*, *Klhl41*, *Rnf217*, *Rnf115*, *Rnf114*, *Rnf123*) (Figure 4), suggesting an increased ubiquitination. Conversely, the term Ub-specific processing proteases (FDR = 2.484 × 10^−3^) mostly includes ubiquitin-peptidase genes (*Usp28*, *Usp44*, *Usp30*), as well as one shared E3 ubiquitin-ligase gene (*Rnf123*), along with genes of proteasome regulatory (*Psmd5*, *Psmd7*) and core (*Psmb1*) components (Figure 4). Collectively, these two enriched terms suggest tight regulation of protein degradation.

The Macroautophagy term (FDR = 5.094 × 10^−8^) encompasses genes such as *Ambra1*, an autophagy regulator, and autophagy-related genes (*Atg4a*, *Uvrag*), which beside kinase genes (*Mtor*, *Src*) indicate multiple degradation pathways activity, including mitophagy identified as an additional enriched term (Figure 4). The core genes in Mitophagy-animal term (FDR = 6.834 × 10^−6^) include *Ambra1*, *Src*, *Ubb* (ubiquitin), *Nbr1* (a selective autophagy adaptor), *Usp30* (deubiquitylase constitutively associated with the outer mitochondrial membrane proteins), and *Mfn2* (involved in mitochondrial dynamics and ER mitochondria contact sites). The ABC-family proteins mediated transport term (FDR 2.506 × 10^−3^), including *Abcb8* and *Abcb6* (both involved in mitochondrial transport across its membranes), the Fatty acid metabolism term (FDR = 8.215 × 10^−3^) with *Gpx1* (an antioxidant enzyme), and *Tomm20* (core component of mitochondrial membrane’s protein machinery), suggest alterations in mitochondrial function and may be related to an increased need of mitochondrial quality control.

Enrichment of the Muscle contraction term (FDR = 3.531 × 10^−5^), uniting genes encoding K^+^ and Ca^2+^ channels (*Kcnh2*, *Cacnb4*, *Cacna2d4*, *Cacng1*) with structural proteins of sarcomere (*Tpm2*, *Acta1*) and sarcolemma (*Dysf*, *Cav3*, *Fxyd1*, *Vcl*), may reflect early molecular changes that precede muscle weakness (Figure 4). Here, *Acta1* should be interpreted with caution or even omitted since it did not show up as DEG in any of the datasets (Table S4), and its human ortholog is present in the HSA^LR^ transgene. Adherens junction (FDR = 1.107 × 10^−2^), as the least statistically significant term, includes genes contributing to cadherin-mediated adhesion (*Pvrl3*), actin cytoskeleton anchoring and remodeling (*Vcl*, *Ctnna3*, *Farp2*), as well as intracellular signaling (*Src*).

Proteins encoded by core genes from SIGORA enriched pathways were analyzed in the STRING database, filtering for experimentally validated interactions (confidence ≥ 0.4). This analysis complements SIGORA, revealing experimentally validated interactions among the protein products of SIGORA core genes, divided into four clusters as follows: two pairs (Cav3-Src and Ctnna3-Vcl), a cluster of Ca^2+^-channel proteins (Cacnb4-Cacng1-Cacna2d4), and the largest cluster centered on Ubb with interactions related to the ubiquitin-proteasome system (Supplementary Figure S3).

To determine the influence of HSA^LR^ toxic RNA on direction of individual gene’s expression, DEGs were determined by DESeq2 [17] between HSA^LR^ and wild-type groups using the predefined criterion of an adjusted *p*-value ≤ 0.05 and absolute log base 2 of fold change > 1. The detected DEGs of Hicks et al. dataset (PRJNA1103789) [14] were overlaid with blue module genes and annotated on the corresponding plots (Figure 2B and Figure 4). Examples of DEGs that were also core genes are observed as coordinately up-regulated with roles in K^+^ and Ca^2+^ transport (*Kcnh2*, *Cacnb4*, *Cacna2d4*), ubiquitination and protein folding (*Usp44*, *Rnf115*, *Fbxl13*, *Fbxl16*, *Hspa2*), fatty acid metabolism (*Alox12b*, *Hacd1*), cell adhesion (*Ctnna3*) (Figure 4, Table S4).

**Figure 5:**
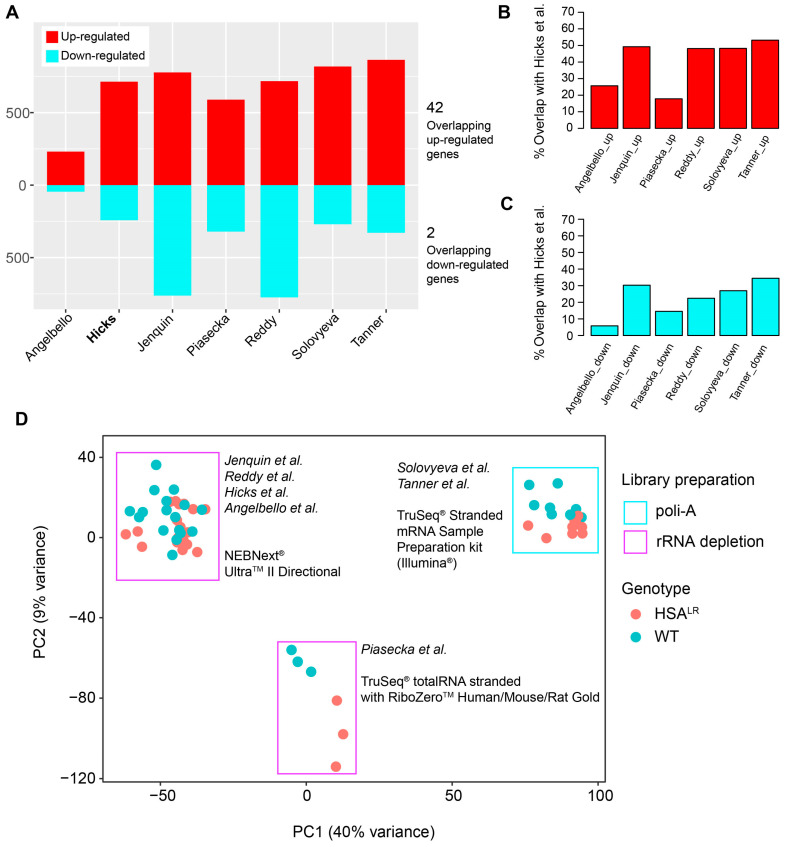
Overview of shared differentially expressed genes (DEGs) among datasets, with the two-dimensional PCA that illustrates the effects of library preparation on the gene expression between datasets. (**A**) An overview of the number of up- and down-regulated genes in the analyzed datasets, with the number of shared up-regulated or down-regulated genes acquired by DESeq2. (**B**) Percentage of shared up-regulated genes and down-regulated genes between the Hicks et al. dataset (PRJNA1103789) and each other analyzed dataset. (**C**) Percentage of shared down-regulated genes between the Hicks et al. dataset (PRJNA1103789) and each other analyzed dataset. (**D**) Two-dimensional PCA performed on merged and variance-stabilized count/expression data from all datasets with annotations for genotype and library preparation.

Next, DEGs were overlapped between seven analyzed datasets (Figure 5A, Tables S1 and S4). Overlapping DEGs showed 42 common up-regulated genes from HSA^LR^ samples compared to their respective wild-type controls, including both protein-coding (*Atp8a2*, *Camk1d*, *Ccdc192*, *Chrna9*, *Cilp*, *Cpne2*, *Cstad*, *Eda2r*, *Hsf2bp*, *Mustn1*, *Myo1a*, *Nrg2*, *Plekho1*, *Plp2*, *Prepl*, *Rrad*, *Runx1*, *S100a4*, *Sbk2*, *Tceal7*, *Trp73*, *Ttc9*, *Uchl1*) and long non-coding RNA (lncRNA) genes (*Atcayos*, *Rian*, *Sp3os*), where the listed ones were also core genes of the blue module (Table S4). There were only two common down-regulated genes in HSA^LR^ datasets (*Hmga2-ps1* and *Vamp1*) (Table S4). These overlapping DEGs are associated with key skeletal muscle cellular components like the sarcolemma, sarcomere or sarcoplasmic reticulum (SR)/endoplasmic reticulum (ER) (*Kcnn3*, *Sln*, *Myo1a*, *Mustn1*, *Uchl1*), mitochondria (*Cstad*, *Vamp1*, *Uchl1*), and the ER/SR specifically (*Sln*, *Uchl1*). In case of *Atcayos*, *Sp3os,* and *Hmga2-ps1*, they are expected to cis-regulate the expression of *Atcay/Nmrk2*, *Sp3*, and *Hmga2*, respectively. The overlap of the DEGs from all datasets (Figure 5A), along with the DEGs from Hicks et al. (PRJNA1103789) [14] with each dataset (Figure 5B,C) shows that gene up-regulation is a more common event.

Finally, to evaluate the possible batch effects on differences in gene expression between the datasets, PCA was performed on the normalized and variance-stabilized counts of all datasets (Figure 5D). It showed the effect of library preparation and separated datasets with 40% variance represented by the first principal component. This effect was considered when comparing DEGs, and led our decision to focus on a single dataset for WGCNA, in addition to sufficient total number of samples for this analysis (minimum is 15 samples).

## 3. Discussion

Despite significant progress in understanding DM1 molecular pathogenesis, the knowledge about downstream effects of toxic RNA on both cellular and systemic levels remains fragmented. They are often studied in isolated experiments with a focus on single mechanism/process, neglecting a more global picture important for the better understanding of complex systems [18]. This exploratory study is based solely on transcriptomic data and presents the first robust gene co-expression network analysis in the most commonly used DM1 mouse model—HSA^LR^ that expresses toxic RNA and mirrors skeletal muscle pathology. We identified prominent transcriptomic changes in 16-week-old mice prior to development of muscle weakness, which typically emerges around 24 weeks with reduced number of satellite cells and myofibers [8]. Using a single Hicks et al. RNA-seq dataset (PRJNA1103789) [14], our analysis captured transcriptomic alternations in multiple processes involved in protein degradation and muscle contraction (Figure 4), indicating that tight regulation of protein homeostasis on which muscle mass and function depend, could be impaired before the onset of muscle weakness.

Coordinately expressed genes in the enriched term Protein processing in ER are mainly involved in ERAD, a quality control mechanism in the protein secretory pathway that helps clear terminally misfolded proteins from ER lumen by directing them toward ubiquitination and proteasome-mediated degradation in the cytoplasm [19,20]. In our results, *Dnajb11* and *Edem3* core genes are involved in selecting misfolded proteins in ER lumen for translocation to the cytoplasm. DNAJB11, a heat shock family 40 chaperone, binds soluble misfolded ER proteins for BiP (HSPA5)-mediated degradation [21], while EDEM3 participates in glycoprotein quality control, where it recognizes proteins stuck in calnexin/calreticulin folding cycle targeting them to ERAD [20]. The core genes also involve *Rnf185* coding ERAD-related E3 ubiquitin ligase localized at the ER membrane, and *Nploc4* whose protein product is part of p97/VCP-NPLOC4-UFD1 complex responsible for extracting ubiquitinated misfolded proteins from ER membrane and delivering them to proteasome [22]. Heat shock 70 family chaperones (represented among core genes by *Hspa1l* and *Hspa2*) recognize defective proteins in the cytoplasm, forming one of the quality control checkpoints for ERAD [20]. Another mechanism of protein quality control in ER is the unfolded protein response, activated when misfolded proteins accumulate in the ER and serving as ERAD back-up [19]. Human DM1 myotubes showed increased mRNA and protein levels of CHOP [23], a human homolog of *Ddit3*, which is an unfolded protein response gene and an ER and cellular stress induction marker [19]. Although it is one of the core genes, *Ddit3* is not co-expressed with any other hallmark genes of unfolded protein response (e.g., *Hspa5*, *Xbp1*, *Eif2ak3*, *Ire1 or Ern1*, *Atf4 or Atf6*) [24] and neither is up-regulated in any of analyzed datasets (Table S4), making the unfolded protein response and ER stress less probable in HSA^LR^ mice at age of 16 weeks. These results may imply that prior the muscle weakness onset, ERAD successfully clears misfolded proteins, alleviating ER stress [19] that has been observed in DM1 patient muscle biopsies and thought to be involved in muscle wasting [25].

The second most significantly enriched term highlights alternation in ubiquitin-proteasome system (UPS) in HSA^LR^ mice. Most myofibrillar proteins are degraded through UPS, which if dysregulated can promote muscle wasting, myofiber degeneration, and muscle weakness [26]. In addition, UPS interacts with ER membrane and degrades secreted proteins that are targeted by ERAD [20]. Identified core genes include ubiquitin-ligases, considered as one of the causes of muscle mass decrease when upregulated [27], proteasomal subunits, and ubiquitin-hydrolase genes. Although HSA^LR^ mice do not yet exhibit muscle weakness at 16 weeks of age, these early transcriptomic changes are in line with increased proteasome activity described in DM300 mice (carriers of ~550 repeats) between 3 and 10 months of age when progressive muscle weakness appeared [28], and in DMSXL mice (carriers of >1000 repeats) at 4 months of age when muscle weakness had already developed [29].

The next protein degradation pathway highlighted in our results is autophagy. It is the second major proteolytic system, extending to degradation of dysfunctional cell components (macroautophagy), such as mitochondria (mitophagy), with the main activation factor being nutrient starvation [26]. Multiple core genes mark the initiation phase of autophagy, where they are involved in the regulation of Beclin1-Vps34 complex (*Ambra1*, *Uvrag*), or acting as cargo degradation receptors of ubiquitinated substrates for autophagy (*Nbr1*) [30]. In line with our findings, HSA^LR^ mice have increased the expression of autophagic and mitophagic proteins and increased tagging of damaged organelles (including mitochondria), likely due to an impaired capacity for protein degradation under basal conditions [31,32]. Furthermore, HSA^LR^ has reduced levels of AMPK activation (an event that activates catabolic pathways such as autophagy) [32], and mild changes in autophagic markers, and also shows AMPK- and mTORC1-independent mechanisms contributing to autophagy [32]. However, in the context of DM1 there are conflicting results about the status of AMPK depending on the model used [18]. Interestingly, there is a notion that autophagy could be a source of energy for the activation of quiescent satellite cells emphasizing its importance for the DM1-affected adult skeletal muscle progenitor cells [33]. Increased autophagic flux along with abnormal activation of UPS, is related not only to muscle weakness, but also atrophy in DM1 [32], a symptom that is not recapitulated by HSA^LR^ mice [7,8]. A series of events described in DM1 cells, but not explored in HSA^LR^, causes the emergence of UPS activity and autophagy, and starts from the STAU1 stabilization, followed by PTEN activation and down-regulation of AKT signaling [18,34]. In addition, in UPS-deficient mice, autophagy is enhanced, and satellite cell function impaired [26], while in myocytes autophagy accounts for 40% of degradation of long-lived proteins with an ability to become a backup for UPS [30]. Balanced autophagy is essential for skeletal muscles: basal activity clears damaged mitochondria and sustains satellite-cell fitness, whereas both excessive autophagy (driving atrophy and apoptosis in some myopathies) and deficient autophagy (in aging or sarcopenia) can culminate in muscle cell death and weakness [35]. Taken together, in skeletal muscles both proteolytic systems are connected [36], and their crosstalk should be explored in DM1.

Mitochondrial dysfunction is proposed to be a contributor to DM1 phenotype in patient skeletal muscles due to severe downregulation of mitochondrial transcription, protein abundance, and respiration [37]. By interpreting autophagy-related results at the level of individual core genes and their features, we identify a co-expression of genes linked to mitophagy suggesting their coordinated regulation. Among these, *Mfn2*, *Ambra1*, and *Usp30* emerged as key components of mitochondrial quality control mechanisms (Figure 4). *Mfn2* is involved in regulating mitochondrial quality control and fusion [38], while *Ambra1* participates in skeletal muscle mitophagy and mitochondrial function, while also regulating the activity of both cell proliferation and autophagy machineries [39]. *Usp30*, a deubiquitinase, regulates mitochondrial dynamics by modulating turnover of outer membrane proteins such as Mfn1 or Mfn2, and its inhibition promotes mitochondrial fusion [40]. This co-expression suggests a known regulatory triad in which USP30-mediated deubiquitination stabilizes Mfn2 to suppress mitophagy, while AMBRA1 provides a compensatory mechanism to restore mitochondrial clearance [41], indicating a dynamic balance between fusion, degradation, and autophagy which could be relevant for mitochondrial integrity in the early DM1 pathology.

Furthermore, mitophagy depends on UPS, as ubiquitination of proteins such as Mfn2 or TOM20 promotes fission and the removal of mitochondria by autophagosomes [36]. The co-expression of ER and mitochondrial genes may reflect adaptations at the level of mitochondria-associated membranes (specialized regions of ER membrane), which facilitate functional coupling of the two organelles with proteins such as Mfn2 or TOM20 that mediate calcium exchange and coordinate mitochondrial dynamics with ER homeostasis [42]. Interestingly, MFN2 has been found to play fusion-independent role, interacting with proteasome and cytosolic chaperones in prevention of newly synthesized protein aggregation [43]. Similarly, *Hspa1l* and *Rnf185* orthologs participate in mitochondrial protein quality control and selective mitophagy, respectively [44,45]. While these findings suggest a potential role for mitochondrial–autophagy crosstalk in maintaining proteostasis, they remain correlative and require experimental validation to establish a causal relationship in early DM1 muscle pathology. Notably, in the context of muscle wasting the disruption of mitochondrial network seems to be crucial for the muscle atrophy program, and it has been shown that expression of fission machinery in mice is sufficient to cause muscle wasting [27]. However, HSA^LR^ mice when compared to patients do not show a decrease in mitochondrial protein abundance between the age of 3 and 6 months [31].

Beyond proteostasis, transcriptomic changes reflect possible alterations in muscle contractile function, but these results can also be traced back to UPS and autophagy. A core gene for calcium-/calmodulin-dependent kinase gene (*Camk2d*) has two isoforms and does not show major splicing changes in HSA^LR^ mice or DM1 cells [46]. However, in cardiomyocytes the increased activity of its cytoplasmic form reduces contractility and alters Ca^2+^ handling [47]. Interestingly, *Kcnh2*, which is also an up-regulated potassium voltage-gated channel gene in Hicks et al. dataset (PRJNA1103789) [14], is shown to be up-regulated in atrophic mice, where its activity modulates ubiquitin proteasome proteolysis in *gastrocnemius* muscles [48]. Among core genes, several voltage-gated calcium channel genes (*Cacnb4*, *Cacna2d4*, and *Cacng1*) along with *Ddit3* may be related to stress due to the accumulation of unfolded proteins in the ER lumen [23]. Ca^2+^ is released from the SR after depolarization of the sarcolemma, but is also known to be involved in proliferation, apoptosis, and protein folding, as chaperones are calcium-binding [23]. Human DM1 myotubes showed uncoupling of the ER/SR Ca^2+^ store and voltage-induced Ca^2+^ machinery [23]. Under dysregulation of calcium cycling dynamics, Ca^2+^ transfer from the ER to the mitochondria can lead to an overload of the mitochondrial matrix, triggering a permeability transition and the collapse of the mitochondrial membrane potential, an increase in ROS, and the release of pro-apoptotic factors [49]. In this regard, we detected two core genes, *Alox12b* and *Gpx1*, both related to oxidative stress response [50,51]. If mitochondria are defective, increased ROS can promote myofiber degeneration by activating AMPK and promoting autophagy as well as UPS degradation [36]. Skeletal muscles do not have any cell–cell junctions, therefore genes belonging to Adherens junction term (Figure 4) should be regarded as early signs of possible cell–matrix cytoskeletal remodeling preceding weakness and involving genes such as *Vcl* [52].

To complement network and pathway analyses, we explored differential expression results across multiple publicly available RNA-seq datasets from untreated HSA^LR^ muscle tissues. This is the first study to compare differential gene expression analysis output across multiple HSA^LR^ datasets, which ensured higher confidence of obtained DEGs. Through the analysis of these datasets, we uncovered frequently occurring DEGs that were largely up-regulated. We identified a common up-regulation of two lncRNAs *Rian* (*AB063319*) and *Atcayos. Rian* expression is dynamic across development and aging [53], while *Atcayos* is up-regulated during the rapid growth stage of satellite cells [54]. In this context, it is important to consider the progressive reduction in muscle strength, along with impaired muscle regenerative capacity in HSA^LR^ [8]. Here, 12-week-old mice show elevated Pax7 expression, suggesting active satellite cells and preserved regeneration, while 24-week-old mice exhibit fewer nuclei below basal lamina, lower amount of Pax7^+^ cells and myofibers, indicating impaired regeneration [8]. Furthermore, *Atcayos* is an antisense transcript from the locus of *Atcay* gene, which is important for acetylcholine signaling [55,56]. *Atcay* is co-localized with mitochondria and plays a role in spatial positioning of mitochondria in neurites [57]. Since lncRNAs are not included in gene ontologies or metabolic pathways databases, but could be regulating genes involved in those terms/pathways, it would be useful to consider them for further research as factors shaping the DM1 molecular pathology. In closing, there are several commonly up-regulated genes which relate to muscle function and therefore DM1 pathology. For example, sarcolipin (*Sln*) is up-regulated in atrophic muscles and may contribute to promoting oxidative metabolism under conditions of metabolic stress [58]. The up-regulation of pre-mRNA of *Sln*, a ubiquitin-hydrolase gene *Uchl1*, as well as ectodysplasin A2 receptor (*Eda2r*) has already been shown by competitive RT-PCR analysis [59]. Additionally, a calcium-dependent protein copine II (*Cpne2*) gene and *Uchl1* have confirmed the up-regulation of protein expression by immunoblot in HSA^LR^ mice [59]. Lastly, the shared down-regulated gene *Vamp1* coding for synaptic vesicle-associated integral membrane protein has a role in Ca^2+^-triggered neurotransmitter release at the mouse neuromuscular junction, where it is also crucial for its efficacy [60]. However, it should be noted that there is no toxic RNA in neurons of HSA^LR^ mice, which limits the representation of neuromuscular junction changes in DM1. These results coming from multiple datasets emphasize complex structural and functional players of early DM1 transcriptome, considering the age range (5-16 weeks) of analyzed HSA^LR^ mice RNA-seq data (Table S1).

The main limitations of this study are that it is exploratory and hypothesis-generating, based solely on transcriptomic data, and it relies on gene-level without considering splicing isoform level. Nevertheless, the results provide a coherent and detailed transcriptomic overview, with discussed key pathways prioritized by enrichment significance. The absence of wet-lab validation represents another limitation.

The results of this study provide basis for many different wet-lab follow-up experimental validations. We are suggesting a subset of genes, considering their expression patterns and correlation with HSA^LR^ genotype, for functional follow-up experiments. To validate our findings, future studies should validate co-expressed genes such as Dnajb11, Edem3, Rnf185, Hspa1l/Hspa2, Ambra1, Mfn2, Usp30, Drp1, Camk2d, Kcnh2 at the protein level using Western blot (WB) in 16-week-old HSA^LR^ mice without severe DM1 symptoms. Additionally, using quantitative PCR (qPCR) with reverse transcription it would be important to measure the expression of identified lncRNAs *Rian* and *Atcayos* since they are present in all analyzed datasets (aged 5 to 16 weeks) as up-regulated (Table S4). The measurement of these lncRNAs should be quantified for different ages of HSA^LR^ mice to correlate their expression with disease progression. It would be interesting to assess UPS function in 16-week-old HSA^LR^ mouse muscles by measuring proteasome activity using fluorogenic substrates, and by evaluating ubiquitin accumulation or K48-linked ubiquitin chains through WB or immunohistochemistry. Furthermore, monitoring the degradation kinetics of misfolded ER protein reporters can in primary myoblasts or myotubes derived from HSA^LR^ mice enable exploring ERAD efficiency in this system. Additionally, one should consider assessing the accumulation of LC3-II both with and without a lysosomal inhibitor to explore autophagy in muscles of 16-week-old HSA^LR^ mice. Furthermore, mitophagy can be assessed by measuring PINK1 accumulation and Parkin Ser65 phosphorylation by WB, combined with qPCR analysis of mitochondrial DNA to quantify mitochondrial mass. Together, these experiments would provide functional validation of our network-based findings, highlighting molecular events present in early DM1.

In conclusion, our study provides the first system-level view of the HSA^LR^ transcriptome using WGCNA, suggesting coordinated early alterations in proteostasis pathways, including ERAD, UPS, autophagy, mitophagy, and genes linked to muscle contraction. These transcriptomic signatures emerge in the absence of apparent muscle weakness, underscoring their potential role as early markers of DM1 pathology. By integrating co-expression and cross-dataset DEG analysis, we highlight a set of robust candidate pathways that warrant validation at the proteomic and functional levels. Future work should explore how these early molecular changes and crosstalk in protein degradation processes connect to muscle weakness progression and whether they can be targeted for therapeutic intervention. Importantly, testing these candidate pathways jointly in patient-derived samples will be essential to determine their relevance beyond the HSA^LR^ model.

## 4. Materials and Methods

Ten publicly available RNA-seq datasets from skeletal muscles of HSA^LR^ mouse models expressing repeat expansion were obtained from the NCBI Sequence Read Archive (SRA) database. Last search was performed on 13th November 2024. Among these datasets, the age ranged from the 5 to 16 weeks, while there were both proximal (*quadriceps*) and distal (*tibialis anterior*, *gastrocnemius*) skeletal muscle tissues (Table S1).

To systematically compare results between datasets, we relied on identical tools throughout the workflow with slight parameter modification according to dataset properties. First, reads were quality-checked, trimmed, mapped, and quantified by using FastQC (v.0.11.9) [61], Cutadapt (v.3.8) [62], hisat2 (v.2.1.0) [63], and featureCounts (v.2.0.0) [64], respectively. Next, differential gene expression analysis was performed by DESeq2 (v.1.46.0) [17] in the R environment version 4.4.2 [65], using genotype (HSA^LR^ or wild type) as the only factor variable in the design formula. The thresholds for the adjusted *p*-value and the absolute log base-2-fold-change for this analysis were as follows: padj < 0.05, |log_2_FC| > 1. All visualizations were performed in R.

The two-dimensional PCA, performed using the *pca* wrapper function from the M3C package (v.1.28.0) [66], on the input in the form of merged counts from all datasets converted into a matrix of variance-stabilized values using DESeq2’s *varianceStabilizingTransformation* function, was used to obtain a general overview of various biological and/or technical effects on gene expression in all data used. For Hicks et al. dataset (PRJNA1103789), PCA was performed individually for variance-stabilized counts using DESeq2’s built-in *plotPCA* function.

To detect co-expression patterns in Hicks et al. HSA^LR^ dataset [14], the weighted gene co-expression network was constructed using the WGCNA package (v.1.73) [13]. The network was built with a soft-thresholding power of 4 by using the *blockwiseModules* function (scale-free topology fit index R^2^ = 0.82). Key parameters included an increased block size (30,000) to ensure a single-block analysis, unsigned network type, unsigned topological overlap matrix (TOM), merge cut height of 0.25 (to favor more distinct modules), and enabled parameters for Partitioning Around Medoids (PAM) clustering with pamRespectsDendro = TRUE to preserve hierarchical clustering of gene dendrograms. These settings were selected to balance network robustness and biological interpretability.

Module-trait relationships were assessed using Pearson correlations between module eigengenes (i.e., their first principal components) and the genotype (1 for HSA^LR^ and 0 for wild-type). For each gene, gene significance (GS) was defined as the Pearson correlation between gene expression and the binarized genotype, and module membership (MM) was defined as the correlation between gene expression and the corresponding module eigengene. Although the network was unsigned and modules may contain both positively and negatively correlated genes, we specifically focused on genes with positive GS and positive MM (with respective *p*-values ≤ 0.05), reflecting those whose expression level positively correlates with HSA^LR^, as well as the module itself, i.e., the module eigengene. This subset of genes is co-expressed and associated with HSA^LR^ genotype.

Over-representation analysis (ORA) for core genes of the WGCNA blue module that were positively associated with HSA^LR^ genotype (GS and MM > 0.8; *p*-value ≤ 0.05) was performed in Metascape (v.3.5.20250101) [67], with enrichment *p*-value < 0.05, gene overlap > 3, enrichment > 1.5, and the following database selection: Gene Ontology (GO): Biological Processes, Molecular Functions, and Cellular Components, Kyoto Encyclopedia of Genes and Genomes Pathways (KEGG), and Reactome. The non-redundant ‘summary’ terms that have FDR (i.e., q-value) ≤ 0.05 from ORA results were plotted using the ggplot2 package [68]. Significant over-representation (SIGORA) of pathway gene-pair signatures for the same group of genes was performed in package sigora (v.3.1.1) [16] in R environment, using KEGG and Reactome databases with FDR cutoff 0.05. Visualization of these results was performed using igraph package (v.2.1.4) [69].

STRING (v.12.0) [70] was queried using core genes from enriched pathways of SIGORA to acquire information on experimentally validated protein interactions. Network type was set to full STRING network, active interaction sources to ‘Experiments’, minimum required interaction score to medium confidence (0.4), while the network nodes were hidden and edges set to depict interaction score.

## Data Availability

The RNA sequencing datasets that were analyzed are available through the NCBI Sequence Read Archive (SRA), https://www.ncbi.nlm.nih.gov/sra/ (accessed on 13 November 2024).

## Supplementary Materials

The following supporting information can be downloaded at https://www.mdpi.com/article/10.3390/ijms262110793/s1, references [11,12,14,71,72,73,74] are included.

## Abbreviations

The following abbreviations are used in this manuscript:

DM1	Myotonic Dystrophy Type 1
WGCNA	Weighted Gene Co-expression Network Analysis
ER	Endoplasmic Reticulum
SR	Sarcoplasmic Reticulum
PCA	Principal Component Analysis
GS	Gene Significance
MM	Module Membership
ORA	Over-representation Analysis
FDR	False Discovery Rate
SIGORA	Significant Over-representation Analysis
DEG	Differentially Expressed Gene
ROS	Reactive Oxygen Species
UPS	Ubiquitin-Proteasome System
ERAD	ER-Associated Degradation
qPCR	quantitative Polymerase Chain Reaction
