# Supplementary material for: HSALR Mice Exhibit Co-Expression of Proteostasis Genes Prior to Development of Muscle Weakness
Source: Int J Mol Sci. 2025 Nov 6;26(21):10793. doi: 10.3390/ijms262110793 (PMC12608036; doi:10.3390/ijms262110793)
Supplement: Supplementary file 1 [file ijms-26-10793-s001.zip › Supplementary_Figures.pdf]

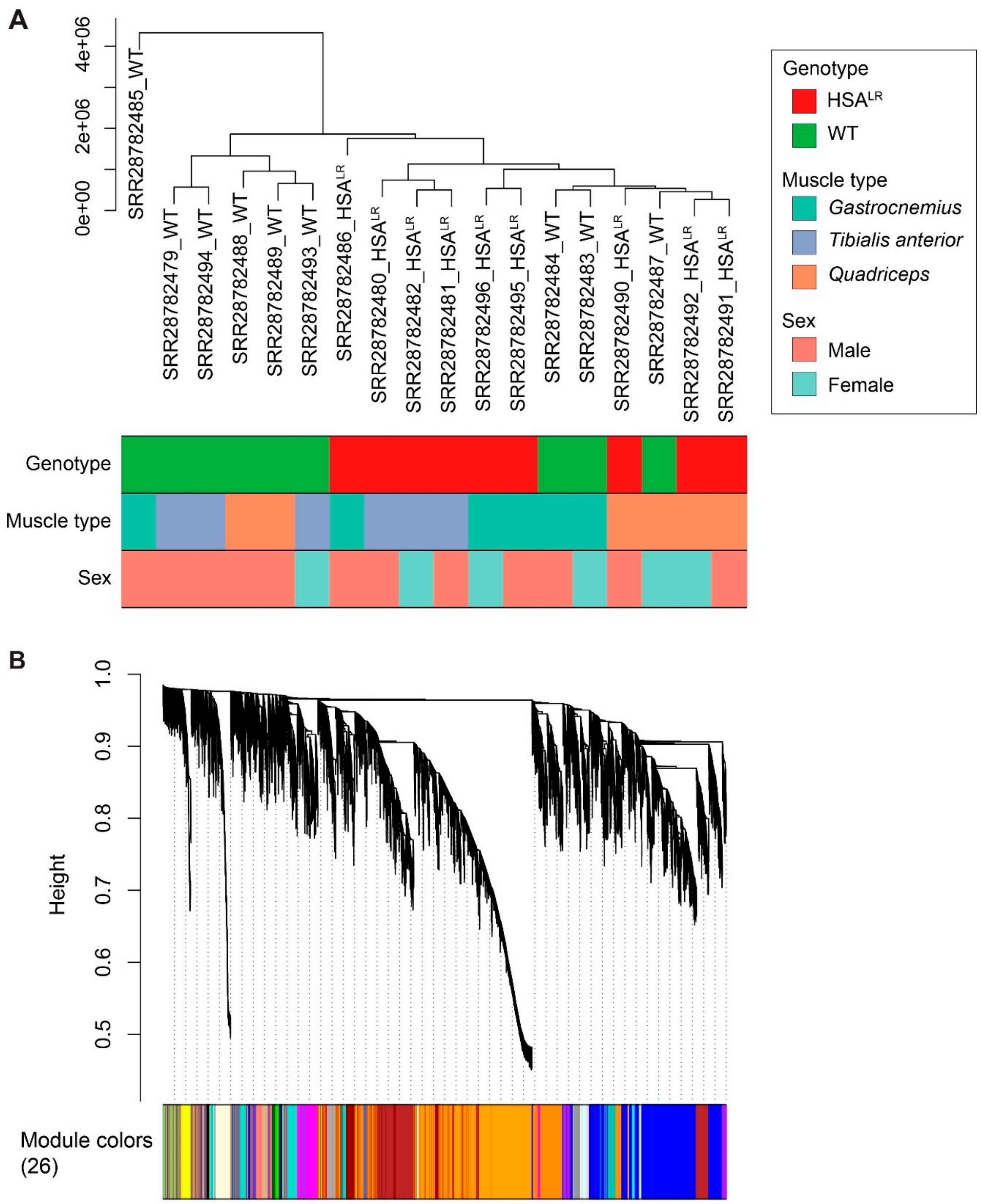

Supplementary Figure S1. Weighted Gene Co-Expression Analysis (WGCNA) sample and gene clustering on Hicks et al. (PRJNA1103789) dataset. (A) Sample clustering performed as a standard step of WGCNA on normalized counts of this dataset with annotations for genotype, muscle type and sex. One outlier (SRR28782479_WT) had slightly lower read assignment in featureCounts, but was considered in the analysis since it did not affect the final output when excluded. **(B)** Gene clustering dendrogram with module color assigned by WGCNA. A total of 26 modules was identified.

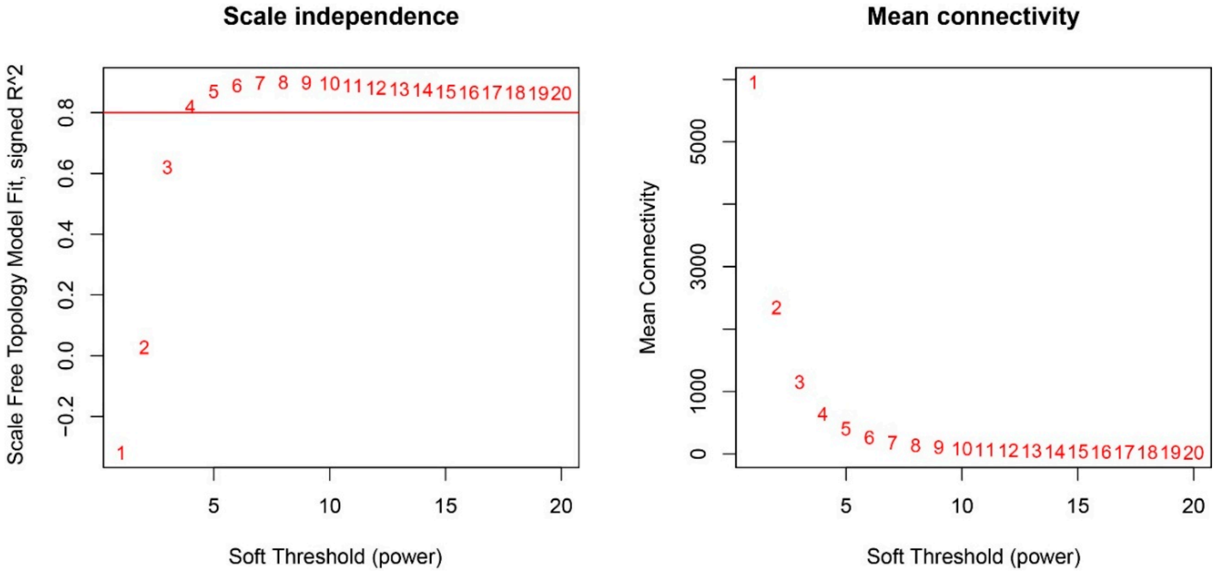

Supplementary Figure S2. The chosen soft-threshold of 4 was the lowest value at which the network approximately fits a scale-free topology for Hicks et al. (PRJNA1103789) dataset. The plot on the right shows mean network connectivity depending on soft-threshold.

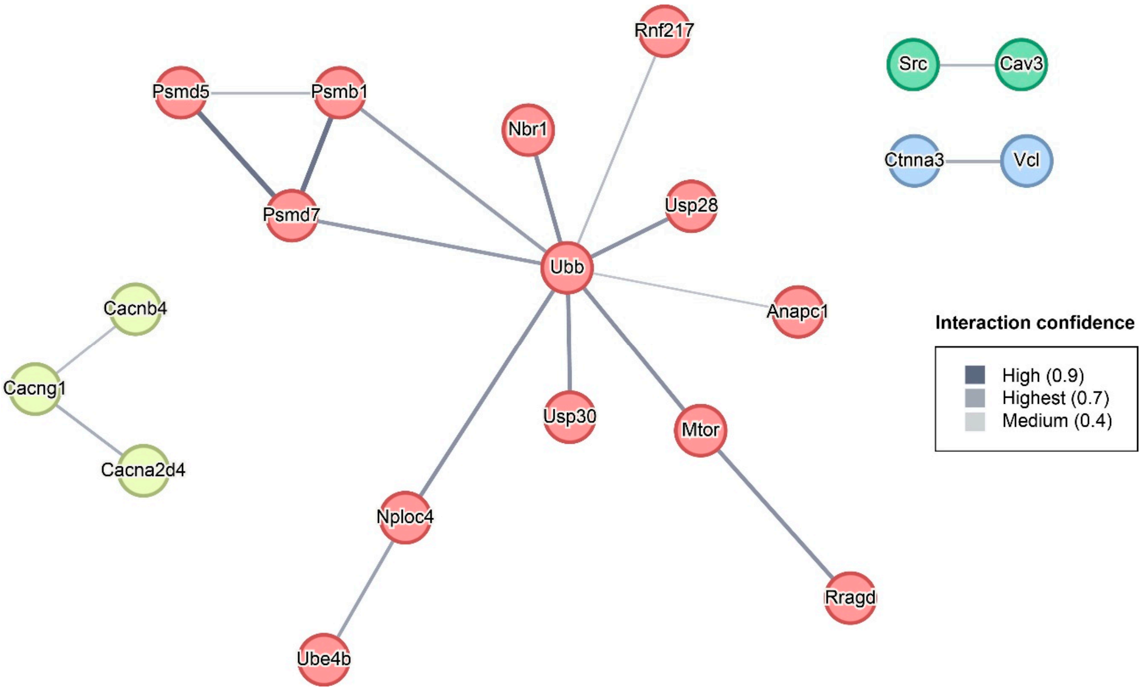

Supplementary Figure S3. STRING network of experimentally validated interactions of core genes from SIGORA enriched pathways reveals four clusters. STRING database was queried using 67 genes from SIGORA enriched pathways and filtered for experimentally validated interactions with medium confidence (≥0.4). Highest confidence interactions were revealed between proteasome subunit proteins (Psmd5, Psmd7, Psmb1), while the whole cluster around Ubb represents ubiquitin-proteasome system. The smaller yellow cluster consisted of calcium voltage-gated channel subunits, while the two pairs of proteins Ctnna3-Vcl and Src-Cav3 have confirmed interactions in heart fascia adherens and caveolin-mediated endocytosis, respectively.
